# Comparability between insecticide resistance bioassays for mosquito vectors: time to review current methodology?

**DOI:** 10.1186/s13071-015-0971-6

**Published:** 2015-07-07

**Authors:** Henry F Owusu, Danica Jančáryová, David Malone, Pie Müller

**Affiliations:** Epidemiology and Public Health Department, Swiss Tropical and Public Health Institute, Socinstrasse 57, PO Box, CH-4002 Basel, Switzerland; University of Basel, Petersplatz 1, CH-2003 Basel, Switzerland; Innovative Vector Control Consortium, Liverpool School of Tropical Medicine, Pembroke Place, Liverpool, L3 5QA UK

**Keywords:** Mosquitoes, Insecticide, Resistance, Susceptibility, CDC bottle assay, WHO susceptibility assay, Bioassay

## Abstract

**Background:**

Insecticides play an integral role in the control of mosquito-borne diseases. With resistance to insecticides on the rise, surveillance of the target population for optimal choice of insecticides is a necessity. The Centers for Disease Control and Prevention (CDC) bottle assay and the World Health Organization (WHO) susceptibility test are the most frequently used methods in insecticide resistance monitoring. However, the two bioassays differ in terms of insecticide delivery and how insecticide susceptibility is measured. To evaluate how equivalent data from the two assays are, we compared the two methods side-by-side.

**Methods:**

We did a literature search from 1998 to December 2014 to identify publications that performed both assays on the same mosquito population and compared the results. We then tested the WHO and CDC bioassays on laboratory strains of *Aedes aegypti*, *Anopheles stephensi*, *An. gambiae* and *An. arabiensis* with different insecticide resistance levels against permethrin, λ-cyhalothrin, DDT, bendiocarb and malathion. In addition, we also measured the relationship between time-to-knockdown and 24 h mortality.

**Results:**

Both published data and results from the present laboratory experiments showed heterogeneity in the comparability of the two bioassays. Following their standard procedures, the two assays showed poor agreement in detecting resistance at the WHO cut-off mark of 90 % (Cohen’s κ = 0.06). There was better agreement when 24 h mortality was recorded in the CDC bottle assay and compared with that of the WHO susceptibility test (Cohen’s κ = 0.5148). Time-to-knockdown was shown to be an unreliable predictor of 24 h mortality.

**Conclusion:**

Even though the two assays can detect insecticide resistance, they may not be used interchangeably. While the diagnostic dose in the WHO susceptibility test does not allow for detecting shifts at low or extreme resistance levels, time-to-knockdown measured in the CDC bottle assay is a poor predictor of 24 h mortality. Therefore, dose–response assays could provide the most flexibility. New standardized bioassays are needed that produce consistent dose–response measurements with a minimal number of mosquitoes.

## Background

Since the resistance of insects to insecticides was first described by Melander in 1914 [1], it has emerged as a major topic for research and discussion in public health because its presence in disease vectors is one of the major obstacles to the reduction of the burden of vector-borne diseases in endemic countries. Over the last decades, resistance of mosquito vectors to insecticides has increased significantly [2, 3] and continues to pose a great threat to the success of chemical control interventions and the control of mosquito-borne disease as a whole. It is known to be present in nearly two thirds of the countries with on-going malaria transmission, in all major vector species and to all available classes of insecticides [4]. Resistance has been detected in at least one insecticide in use for the control of malaria in not less than 64 countries where malaria is endemic [5].

Efforts to curb the threat of insecticide resistance are being scaled up, with one of the most recent steps being the launch of the Global Plan for Insecticide Resistance Management in Malaria Vectors (GPIRM) by the World Health Organization (WHO) [4]. The GPIRM strongly advocates incorporation of insecticide resistance management measures into every vector control programme, even in the absence of resistance. Of utmost importance to these measures is the reliability of the data generated from monitoring and evaluation activities and this is further emphasised by the five pillars outlined in the collective strategy against insecticide resistance in the GPIRM. The acquisition of data largely depends on susceptibility bioassays. The data from these assays are relied on to provide information on the impact of resistance on current interventions and vice versa, leading to an informed choice on strategies to adopt in prevention and management. Although the link between bioassay data and epidemiological failure of a control programme is not well established [6], insecticide susceptibility bioassay data are an indicator if targeted mosquitoes respond well to the insecticides in use [7]. Unfortunately, it is common to find inconsistent testing and reporting of resistance in published data [6], which could be due to several factors including the choice of assay.

The standard bioassay for the detection of resistance in mosquito vector populations is the WHO susceptibility test [8]. This is a direct response-to-exposure analysis that uses discriminating concentrations to distinguish between resistant and susceptible mosquito populations [8]. In 1998, an alternative to the WHO assay, a time-mortality analysis known as the Centers for Disease Control and Prevention (CDC) bottle assay, was developed by Brogdon and McAllister [9]. Currently, both assays are being widely used both in the field and in the laboratory for routine monitoring of resistance. Though both assays measure insecticide susceptibility, they differ in several aspects and as a result, each has some advantages over the other [10]. The CDC bottle assay might be chosen over the WHO susceptibility assay because it can be carried out without the need of ordering specialised equipment, which may be difficult to procure. The assay also provides the convenience of evaluating different concentrations of an insecticide as well as the possibility of using synergists, which allows for fast and inexpensive assessment of metabolic resistance mechanisms.

While the CDC bottle assay may be customised to individual needs, the WHO susceptibility assay is less prone to problems with quality assurance and control. Test kits and insecticide-impregnated papers may all be obtained from a centralised source, thus reducing the introduction of variability. This also makes it easier to trace the source of problems, should they arise. It is also easier to separate dead and live mosquitoes, allowing further analysis on these two groups.

The growing rate of insecticide resistance emphasises the need for data of good quality, and with the widespread use of the two bioassays for resistance detection, we set out to assess the extent to which they are interchangeable and how much time-to-knockdown is a proxy for 24 h mortality. First, we reviewed published data to evaluate the existing evidence of comparability between the two assays. We subsequently compared the two assays side-by-side, following their published guidelines on characterised laboratory mosquito strains. Finally, we carried out laboratory experiments to investigate the relationship between time-to-knockdown and eventual 24 h mortality, which are the basic end points of the CDC bottle assay and the WHO susceptibility assay, respectively.

## Methods

### Literature review

We conducted a literature search in compliance with the PRISMA (Preferred Reporting Items for Systematic Reviews and Meta-Analyses) guidelines [11] and systematically searched the National Library of Medicine’s PubMed, ISI Web of Science, EMBASE and the Cochrane library to identify publications that performed susceptibility assays on adult mosquitoes. We searched studies that were published between 1998, when both bioassays have been in use, and December 2014. We used the search terms “Culicidae” in combination with “insecticide resistance” or “insecticide susceptibility”. Our interest was in publications that conducted both the CDC bottle and WHO susceptibility assays on the same mosquito populations following their standard protocols. We therefore excluded publications which only collected susceptibility data on larvae, those that performed neither the CDC nor the WHO assay and those that performed only one of the two assays.

### Mosquito strains

A total of seven laboratory maintained strains were used in the experiments (Table 1). Mosquitoes were reared at temperature and relative humidity ranges of 26–28 °C and 60–74 %, respectively, in a 12:12 h day:night regime. All the strains listed above were used in the evaluation of the relationship between time-to-knockdown and 24 h mortality except JHB and NDJA which were excluded from the comparison between the CDC and WHO assays due to non-existence of standard concentrations for testing *Culex spp* and insufficient mosquito numbers in NDJA. The KISUMU1 and JHB strains were established in our insectary in 2011. The STI and ROCK strains were also established in 1971 and 1978, respectively. All other mosquitoes were acquired as eggs and the emerging adults were then used for the assays.

**Table 1:** Mosquito strains used in the insecticide susceptibility assays

Species	Strain	Source	Resistance status	Resistant to	Known resistance mechanism
*An. gambiae s.s.*	KISUMU1	MR4 (MRA-762)	S		
*An. gambiae s.s.*	VK7	LSTM	R	Pyrethroid and DDT	kdr
*An. arabiensis*	NDJA	LSTM	R	Pyrethroid and DDT	Metabolic
*Cx. quinquefasciatus*	JHB	MR4	R	Pyrethroid and DDT	Not known
*An. stephensi*	STI	LSHTM	R	Pyrethroid and DDT	Suspected metabolic
*An. gambiae*	ZAN/U	MR4	R	DDT	Metabolic
*Ae. aegypti*	ROCK	JHU	R	Reduced susceptibility to DDT	Not known

“*R*” indicates resistant and “*S*” indicates susceptible

*MR4*-Malaria Research and Reference Reagent Resource Center

*LSTM*-Liverpool School of Tropical Medicine

*LSHTM*-London School of Hygiene and Tropical Medicine

*JHU*-Johns Hopkins University

### Insecticides

Five different insecticides, representing the four classes available for public health applications against adult mosquitoes were used: the carbamate bendiocarb, the organochlorine DDT, the organophosphate malathion and the pyrethroids permethrin (25:75 cis:trans) and λ-cyhalothrin. All insecticide solutions used in the CDC bottle assay were self-prepared. Bendiocarb, DDT and malathion were purchased as analytical grades from Sigma-Aldrich® (Switzerland), while permethrin and λ-cyhalothrin were kindly provided by Syngenta Crop Protection AG (Switzerland). All insecticide treated filter papers for the WHO susceptibility tests were sourced from the WHO Pesticide Evaluation Scheme (WHOPES) through Universiti Sains Malaysia based in Penang, Malaysia.

### CDC bottle assay

The preparations, diagnostic doses, exposure time (Table 2) and assay procedure were all as recommended by the CDC guidelines [12]. Prior to performing the assays, 250 ml SIMAX bottles (Kavalierglass Co. Ltd., Czech Republic) were coated the previous evening with the desired insecticide dissolved in acetone. Three to five-day-old non blood-fed female mosquitoes were introduced into the treated bottles and observed for knockdown up to a maximum of 120 min. To allow for the evaluation of the relationship between time-to-knockdown and 24 h mortality, mosquitoes were individually exposed till they were knocked down. Once knockdown occurred, the mosquito was immediately transferred into a small 30 ml plastic beaker. The beaker was then covered with a small piece of cotton mosquito net and labelled with a unique identification number. The mosquito’s id and time-to-knockdown were recorded. Each mosquito was provided with 10 % sugar solution and held for 24 h after which mortality was recorded. Temperature and relative humidity recorded during the laboratory testing ranged from 25.9 to 29.6 °C and 59 to 77 %, respectively.

**Table 2:** Mosquito strains and insecticides used in the WHO susceptibility test and the CDC bottle assays

Strain	Insecticide	Diagnostic concentration		Diagnostic exposure time (min)		Estimated insecticide concentration on surface (μg/cm^2^)	
		WHO (%)	CDC (μg/bottle)	WHO	CDC	WHO	CDC
KISUMU1	permethrin	0.75	21.5	60	30	27.53	0.089
	λ-cyhalothrin	0.05	12.5	60	30	1.84	0.052
	bendiocarb	0.1	12.5	60	30	3.67	0.052
	malathion	5	50	60	30	183.50	0.21
	DDT	4	100	60	45	146.8	0.41
VK7	permethrin	0.75	21.5	60	30	27.53	0.089
	λ-cyhalothrin	0.05	12.5	60	30	1.84	0.052
STI	permethrin	0.75	21.5	60	30	27.53	0.089
	λ-cyhalothrin	0.05	12.5	60	30	1.84	0.052
	bendiocarb	0.1	12.5	60	30	3.67	0.052
	malathion	5	50	60	30	183.50	0.21
	DDT	4	100	60	45	146.80	0.41
ZAN/U	permethrin	0.75	21.5	60	30	27.53	0.089
	DDT	4	100	60	45	146.80	0.41
ROCK	permethrin	0.25	15	60	30	9.18	0.062
	λ-cyhalothrin	0.03	10	60	30	1.10	0.041
	bendiocarb	-	12.5	-	30	-	0.052
	malathion	0.8	50	60	30	29.36	0.21
	DDT	4	75	30	45	146.80	0.31
JHB	permethrin	-	21.5	-	30	-	0.089
	λ-cyhalothrin	-	12.5	-	30	-	0.052
	bendiocarb	-	12.5	-	30	-	0.052
	malathion	-	50	-	30	-	0.21
	DDT	-	100	-	45	-	0.41

Insecticide in μg per cm^2^ was calculated in the CDC assay by dividing the amount in μg per bottle by the estimated surface area of the inner wall of the glass bottle. In the WHO assay, it was calculated based on information provided in the guidelines [25]

### WHO susceptibility test

The WHO susceptibility tests were performed according to the latest published guidelines [8]. Three to five-day-old non blood-fed female mosquitoes were exposed in batches of 24 to 27 individuals to insecticide-treated filter papers at the WHO discriminating concentrations and exposure times [13] (Table 2). After exposure, mosquitoes were transferred back into the holding tube, provided with 10 % sugar solution and kept for 24 h after which mortality was recorded. As per WHO definition, a mosquito was scored in both assays as alive if it was able to fly, irrespective of the number of legs still intact and dead, or knocked down, if immobile or incapable of flying or standing in a coordinated manner [8].

### Data analysis

All data analysis was done in the freely available statistical software package R, version 3.0.2 [14]. The level of significance was set at *α* = 0.05.

For the interpretation of the bioassay results and, in accordance with the current WHO guidelines [8], we applied a 90 % mortality or knockdown threshold as the cut-off value to score resistance. The guidelines for the CDC bottle assay also refer to the WHO definition of resistance [12]. In addition to the 90 % cut-off value, we also compared the two bioassays at 98 % level because, according to WHO, this would be the rate below which resistance is suspected [8].

In the laboratory study, we compared the two tests for agreement in two different ways. First we compared the outcome measures as defined by their protocols (i.e. 24 h mortality in the WHO and knockdown at the diagnostic time in the CDC bottle assay) and called this the “standard comparison”. Secondly, the two assays were compared using 24 h mortalities measured in both assays. We called this comparison the “24 h comparison”. Comparisons were done between the two assays for each mosquito strain and insecticide combination. A single comparison for a single strain and insecticide is here referred to as a “pair”.

Cohen’s Kappa (*κ*) [15] was calculated to quantify the magnitude of agreement between the WHO susceptibility test and the CDC bottle assay, both in the data extracted from the literature search and bioassays conducted in the present study. *κ* accounts for agreement taking place only by chance beyond simple per cent agreement calculations. Its values are interpreted as poor (*κ* ≤ 0), slight (0 < *κ* ≤ 0.2), fair (0.2 < *κ* ≤ 0.4), moderate (0.4 < *κ* ≤ 0.6), substantial (0.6 < *κ* ≤ 0.8) and almost perfect agreement (0.8 < *κ* ≤ 1.0) [16].

In addition to the two end points of percentage mortality and knockdown, we also investigated whether the two assays produced similar patterns in the cumulative number of mosquitoes knocked down as a function of time. For example, if strain A was knocked down quicker than strain B against permethrin in the WHO susceptibility assay, would the same be observed in the CDC bottle assay, and vice versa? If this were the case, it would suggest that the two bioassays yield similar outcomes qualitatively despite their differences, including insecticide concentrations. For their comparison, the knockdown curves for all mosquito strains were analysed using the Kaplan-Meier survival function [17, 18]. First it was determined, separately for the two bioassays, whether there was any difference between the strains tested for a particular insecticide. If so, the order in which the insecticide knocked down the mosquitoes was compared between the two assays. Lines which appeared to overlie or were very close to one another were further analysed for differences.

Finally, the relationship between 24 h mortality and time-to-knockdown was investigated by generalised linear mixed-effects models (GLMM) with a logit link function. We used data from tests against permethrin, λ-cyhalothrin and DDT because of very high levels of susceptibility in all the strains to malathion and bendiocarb. Owing to differences in the action times of insecticides and the different susceptibility levels of the strains, we analysed data from the different strains separately for each insecticide. Twenty-four hours mortality was predicted by time-to-knockdown, with the day of testing included as a random effect term to account for correlations within the same day. The GLMMs were modelled using the R package lme4 [19, 20].

## Results

### Literature review

The database search pulled out a total of 6536 records which were systematically screened to remove publications that were not relevant to our study. After the removal of duplicates, 3773 eligible records were reviewed. Nine publications in which the two assays were performed on the same mosquito population were identified. Results from the identified studies showed mixed outcomes. The agreement ranged from poor to perfect. Three publications had perfect agreement in the strains tested, two showed poor agreement and the rest were moderate to substantial (Table 3).

**Table 3:** Comparison between WHO susceptibility and CDC bottle assay data from the literature search

Study	Country	Species tested	Insecticides tested	CDC diagn. dose used	Pairs (*N*)	*κ*	Quoted protocol
Perea et al. [21]	Peru	*An. albimanus*	deltamethrin	Determined by authors	2	1.00	1998
Hargreaves et al. [24]	South Africa	*An. funestus*	permethrin	Determined by authors	21	−0.02	1975
Matowo et al. [22]	Tanzania	*An. arabiensis*	permethrin	Determined by authors	2	1.00	1981
Aïzoun et al. [10]	Benin	*An. gambiae*	deltamethrin, bendiocarb	CDC recommended	12	1.00	1998
Fonseca-González et al. [32]	Columbia	*Ae. aegypti*	deltamethrin, cyfluthrin, permethrin, etofenprox, malathion,fenitrothion, DDT, λ-cyhalothrin	Determined by authors	96	0.82	1981, 1998
Ocampo et al. [33]	Columbia	*Ae. aegypti*	deltamethrin, λ-cyhalothrin, malathion, fenitrothion, Bendiocarb, DDT	Determined by authors	46	0.55	1981
Fonseca-González et al. [34]	Columbia	*An. darlingi*	deltamethrin, λ-cyhalothrin, fenitrothion, malathion, DDT	Determined by authors	24	0.70	1981, 1998
Fonseca-González et al. [35]	Columbia	*An. nuneztovari*	deltamethrin, λ-cyhalothrin, malathion, fenitrothion, DDT	Determined by authors	24	0.52	1981, 1998
Ochomo et al. [23]	Kenya	*An. gambiae* s.s.	permethrin, deltamethrin, bendiocarb	CDC recommended	3	0.00	1998

### WHO susceptibility test vs. CDC bottle assay from present study

#### Comparison of mortality rates

In the present laboratory study, we had a total of 18 pairs of results available for comparison between the CDC bottle assay and the WHO susceptibility assay. Here, the two assays showed variations in the degree of agreement at the various levels of comparison (Table 4). In the standard comparison, agreement in detecting resistance was only slight at both the 90 % (*κ* = 0.06) and 98 % (*κ* = 0.01) cut off marks. In the 24 h comparison, the agreement improved to moderate at 90 % (*κ* = 0.51) and also at 98 % (*κ* = 0.58).

**Table 4:** Comparison between WHO susceptibility and CDC bottle assay data in the present study

Strain	Insecticide	*N*		% 24 h mortality		% KD at CDC diagn. time	Status			
							90 %		98 %	
		WHO	CDC	WHO (95 % CI)	CDC (95 % CI)		W	C	W	C
ROCK	Permethrin	110	100	96.4 (90.6 - 98.8)	96.0 (89.8, 98.7)	100	S	S	R	S
	λ-cyhalothrin	108	100	99.1 (94.3 - 100)	75.0 (65.6 - 82.5)	100	S	S	S	S
	Malathion	101	100	100 (96.4 - 100)	100 (96.4 - 100)	100	S	S	S	S
	DDT	118	100	67.0 (58.0 - 74.8)	75.0 (65.6 - 82.5)	100	R	S	R	S
KISUMU1	Permethrin	102	100	99.0 (94.1 - 100)	90.0 (82.3 - 94.6)	99	S	S	S	S
	λ-cyhalothrin	108	100	98.2 (93.1 - 99.9)	88.0 (80.0 - 93.1)	100	S	S	S	S
	Malathion	105	100	100 (96.6 - 100)	100 (96.4 - 100)	91	S	S	S	R
	Bendiocarb	107	100	100 (96.6 - 100)	100 (96.4 - 100)	100	S	S	S	S
	DDT	110	100	100 (96.7 - 100)	98.0 (92.5 - 99.9)	99	S	S	S	S
STI	Permethrin	104	102	71.2 (61.8 - 79.0)	52.9 (43.3 - 62.3)	100	R	S	R	S
	λ-cyhalothrin	95	100	32.6 (24.1 - 42.6)	16.0 (10.0 - 24.6)	100	R	S	R	S
	Malathion	110	100	100 (96.7 - 100)	100 (96.4 - 100)	82	S	R	S	R
	Bendiocarb	108	100	100 (96.6 - 100)	100 (96.4 - 100)	100	S	S	S	S
	DDT	104	100	47.1 (37.8 - 56.6)	60.0 (50.2 - 69.1)	99	R	S	R	S
VK7	Permethrin	100	100	78.0 (68.8 - 85.0)	91.0 (83.5 - 95.3)	96	R	S	R	R
	λ-cyhalothrin	49	40	93.9 (82.7 - 98.4)	95.0 (82.4 - 99.4)	100	S	S	R	S
ZANU	Permethrin	103	100	100 (96.5 - 100)	87.0 (78.8 - 92.3)	100	S	S	S	S
	DDT	108	100	96. 3 (90.5 - 98.8)	67.0 (57.3 - 75.4)	100	S	S	R	S

Wilson’s method [31] was used to calculate the confidence intervals in the 24 h mortalities

#### Comparison of knockdown curves

**Fig. 1:**
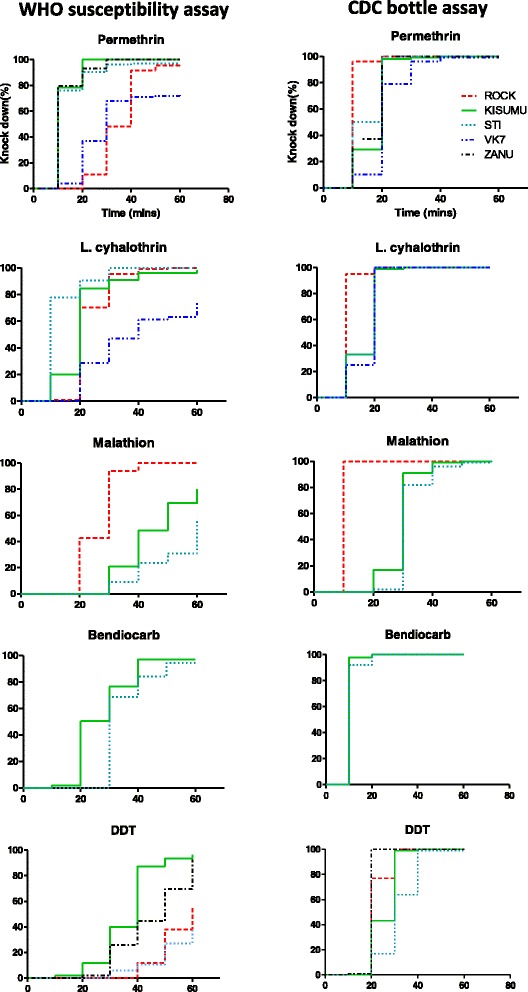
Comparison of cumulative knockdown rates in the WHO susceptibility test (left panels) and the CDC bottle assay (right panels). Knockdown was scored at 10 min intervals

The order and rate of knockdown in the various strains and insecticides was not always the same in both tests (Fig. 1). The survival analysis showed that the order and patterns of the lines of cummulative knockdown of the strains for the various insecticides were all statistically significant in both assays (*p*-values; for WHO: <0.01 for all insecticides, for CDC: <0.01 for all insecticides except bendiocarb, *p* = 0.05). Lines which were very close or overlying also showed statistically significant differences in the rates of knockdown except for STI, KISUMU1 and ZANU tested against permethrin (WHO; *χ*^2^ = 4.2 *p* = 0.121, CDC; *χ*^2^ = 10.8 *p* <0.01) and KISUMU1, VK7 and STI against λ-cyhalothrin (WHO; *χ*^2^ = 126, *p* <0.001, CDC; *χ*^2^ = 0.9, *p* = 0.65).

### 24 h mortality as a function of time-to-knockdown

A total of 2405 mosquitoes from seven strains were tested against five insecticides (Table 1). Time-to-knockdown was a very poor predictor of 24 h mortality. In all the strains tested, it only showed a significant association with 24 h mortality when ROCK was tested against DDT (OR = 0.87, *p* = 0.02, 95 % CI = 0.77–0.98) and VK7 against permethrin (OR = 0.78, *p* <0.001, 95 % CI = 0.68–0.90). Table 5 shows a summary of the odds ratios and significance levels in each strain and insecticide combination.

**Table 5:** Summary of the relationship between time-to-knockdown and 24 h mortality in the various strains given for each insecticide

Strain	Insecticide	N	OR	p	95 % CI
JHB	Permethrin	100	0.99	0.72	0.94 – 1.03
	λ-cyhalothrin	100	0.99	0.59	0.94 – 1.03
	DDT	101	1.0	0.59	0.97 – 1.05
STI	Permethrin	102	1.09	0.28	0.93 – 1.27
	λ-cyhalothrin	100	0.84	0.12	0.66 – 1.05
	DDT	100	0.97	0.29	0.91 – 1.03
ROCK	Permethrin	100	0.67	0.31	0.35 – 1.40
	λ-cyhalothrin	100	1.18	0.26	0.88 – 1.58
	DDT	100	0.87	0.02	0.77 – 0.98
ZANU	Permethrin	100	1.07	0.56	1.35 – 0.85
	DDT	100	0.96	0.64	0.79 – 1.16
VK7	Permethrin	100	0.78	<0.001	0.68 – 0.90
	λ-cyhalothrin	40	0.25	0.14	0.04 – 1.57
	DDT	18	0.94	0.25	0.84 – 1.04
KISUMU1	Permethrin	100	1.13	0.37	0.87 – 1.47
	λ-cyhalothrin	100	0.96	0.73	0.75 – 1.22
	DDT	100	0.84	0.38	0.56 – 1.24

**Fig. 2:**
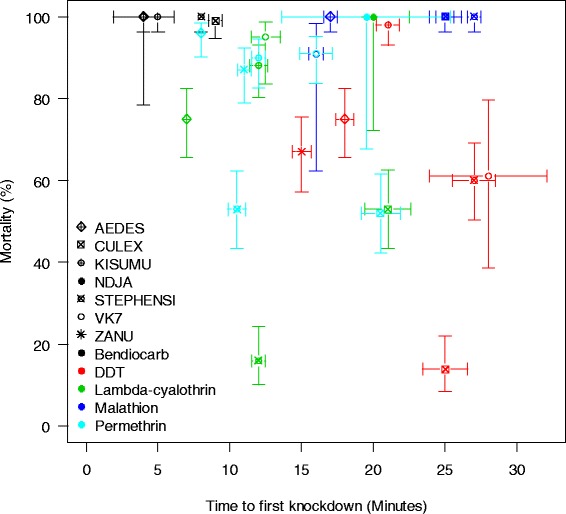
Summary plot showing the relationship between 24 h mortality and the time it takes to knockdown 50 % of the population (KDT_50_) for all strains and insecticides tested in the present laboratory study. Symbols show point estimates and 95 % confidence intervals and colours show the insecticides tested. The confidence intervals for mortality are computed after Wilson [31] and those for KDT using the boxplot function

A plot of the time it takes to knockdown 50 % of the test population (KDT_50_) from the various strains and insecticides against mortality showed no clear pattern, corroborating the results from the GLMM models (Fig. 2). The KDT_50_ values ranged from 4 min in the treatment of ROCK and VK7 with bendiocarb to 28 min in the treatment of VK7 with DDT.

## Discussion

Knowing the insecticide susceptibility or resistance status is key in choosing the appropriate intervention to control any mosquito population. Many factors influence the outcome of insecticide resistance monitoring exercises and the importance of the choice of the assay cannot be overlooked. The results from our study show that although the CDC bottle assay and WHO susceptibility test are mutually used to detect insecticide resistance, the two must not be used interchangeably because the agreement between the two is inconsistent. This could be explained by the fact that the two assays differ in their basic setup. The WHO susceptibility assay, carried out in 125 mm length × 44 mm diameter tubes, measures 24 h mortality by exposing mosquitoes to a known standard concentration for a fixed period of time, usually 1 h. The insecticides are delivered through impregnated filter papers and the standard concentration is twice the lowest concentration that produced 100 % mortality systematically from a baseline analysis of a susceptible strain [8]. Intended to be a simple, easy-to-use assay for field work due to its economical and convenient setup, the CDC assay measures the length of time it takes for an insecticide to “kill” a sample of adult mosquitoes exposed to a known concentration. It is presented in 250 ml glass bottles and the insecticide delivery is by coating the inner walls of the glass bottle [12]. Although in both assays the insecticides are dissolved in acetone, the insecticide carriers differ due to the addition of oil to the mixture in the WHO assay. The insecticide is therefore carried by the oil since acetone is volatile.

From our literature search, the WHO susceptibility assay was the more widely used assay. A notable observation from the search was that the version of the WHO assay protocol quoted as the one followed for the methodology differed between publications, with some recent publications still quoting the 1981 version (Table 3). Even though there have not been a lot of major changes in the procedure in the various versions, there have been updates in some details and recommended concentrations. Despite a lot of studies employing the use of one or the other of the two assays, only nine have used them both on the same population. Aïzoun et al. [10] did a direct comparison of the two tests. While there was high consistency observed in their study, our findings suggest the opposite. Their results might have been influenced by the high susceptibility to bendiocarb and high *kdr* frequency, which resulted in high resistance to deltamethrin in the field populations they used, as opposed to the heterogeneity in our resistance phenotypes. It is also not very clear whether the number of pairs also had an influence on the agreement. Perea *et al*. [21] and Matowo et al. [22] both observed almost perfect agreement with two pairs while Ochomo et al. [23] found a poor agreement with three pairs. On the other hand, Hargreaves et al. [24] also recorded a poor agreement with 21 pairs.

A shortfall of our study was our inability to test all of our strains against all of the present insecticides due to lower numbers available in certain strains. This led to some results being excluded from the comparison as the numbers were considered too low. Moreover, it was not feasible to run all assays simultaneously to exclude daily variations. However, we were careful to maintain equal rearing and testing conditions and randomised test sequences. In addition, we included a random term in the GLMMs to account for correlations within a test day in the analysis of 24 h mortality as a function of time-to-knockdown.

Several factors could have been the cause of the discrepancies observed in the published and present data. It is not clear what impact the concentration used in the CDC assay, whether self-established or CDC recommended diagnostic doses, has on the agreement of the two assays (Table 3). For example, Aïzoun et al. [10] and Ochomo et al. [23] both used the CDC recommended concentrations but had different agreement levels. It could be argued that the low number of pairs (i.e. 3) in Ochomo et al. introduced a bias. Nevertheless, our results, having 18 pairs, still yielded disagreements between the two assays. One explanation might be the difference between the applied concentrations in the two assays. According to WHO [25] the filter papers are prepared such that a treatment at 1 % contains 36.7 μg insecticide per cm^2^. Standard filter papers treated with permethrin, for example, would then contain about 300 times more insecticide per cm^2^ than in the CDC bottle assay (Table 2). But despite the gap in concentrations, the amount of insecticide available to the mosquito in the WHO assay is not clear. These figures are calculated based on the surface area and not the volume of the filter paper, which unlike the glass surface, absorbs the insecticide carrier used in the preparation. This is evident in the fact that during preparation, insecticides are applied on one side of the filter paper, the “right” side, but the “wrong” side also contains enough insecticide to kill mosquitoes. Although concentrations are higher in the WHO assay, this might not be reflected in the insecticide availability due to the method of delivery. This could also explain why the order and rates of knockdown were inconsistent between the two assays in our study. The curves in the CDC assay were steeper, indicating a faster knockdown rate than in the WHO assay. The statistical significance of the Kaplan-Meier survival function also shows that these assays are not only inconsistent in the output of mortality, but also knockdown. With the WHO diagnostic concentrations in use for over a decade [26], it might be time to review them. This could provide a good opportunity to bring the two assays in synchrony. A possible solution to the current inconsistent agreements could be for WHO and CDC to come together to re-calibrate both assays using the same population of mosquitoes. In doing so, the concentrations and exposure times that would provide the same level of mortality can be derived for each assay.

Evidently missing in the CDCs setup is a 24 h holding period. Though this makes the assay rather short and rapid, we believe that some level of resistance, especially metabolic resistance could be missed in the CDC assay due to the lack of a recovery time. This was observed in the results obtained from our laboratory tests, especially in the STI strain (Table 4). At both the 90 and 98 % knockdown threshold *κ* was very low. This may be explained by the fact that the CDC assay scored most of the strains as susceptible due to the high knockdown rates. This increased the agreement in scoring the colony as susceptible but not as resistant. Holding the mosquitoes from the CDC assay for 24 h significantly increased the agreement from poor to moderate due to the recovery in some strains. No holding period in the CDC assay also means that “dead” mosquitoes are recorded only during the period of exposure. According to the WHO guidelines, knockdown is recorded during the exposure period and mortality is recorded 24 h post exposure. Therefore, by applying this definition, the CDC assay only measures knockdown, and the WHO assay measures mortality. This then raises the question of how much 24 h mortality may be explained by time-to-knockdown. From our results, the relationship between the two is rather unreliable, feeding into the long standing question of which is the best way to measure resistance. As previously observed, in the presence of very high resistance, time-response assays have not been very useful [27, 28].

With time-to-knockdown being rather a poor predictor of mortality, a better way of measuring 24 h mortality could be the use of dose–response curves. These curves are dynamic, provide more flexibility and are more informative. With current assays the downside is, however, the requirement of higher mosquito numbers. For example, according to the WHO guidelines, at least 600 mosquitoes would be required, a number often difficult to collect in the field. A single diagnostic concentration (LC_99_) may remain unchanged in the presence of low numbers of homozygous resistant individuals. The LC_50_, however, might shift with growing numbers of heterozygous resistant individuals if the mutation has a co-dominant effect [29]. A comprehensive dose-mortality therefore, would enable the detection of any early indications of emerging resistance. This would also facilitate the comparison of susceptibility over time within or between populations [30]. While being sensitive to low levels of decreased sensitivity, likewise, it would still capture changes at the high end of resistance [27]. Practically, a remaining challenge will be the choice of concentrations at which 24 h mortality is measured in order to make sound dose–response curve estimates.

## Conclusion

This study shows that though the two assays can both successfully detect insecticide resistance, they may not be used interchangeably due to the high level of inconsistencies in the agreement between them. Since a lot of factors go into the results obtained from bioassays, the choice of assay is very important. At a time when the WHO is putting a lot of emphasis on monitoring and evaluation, the choice of the assay should be based on the data and information required rather than which is readily available. Given their various advantages over the other, they will be very beneficial to control programs if their purposes are re-defined and adopted for specific functions in insecticide resistance monitoring and evaluation. With time-to-knockdown being a poor predictor of mortality, turning to dose–response assays could provide the most flexibility as it eliminates the dependence on diagnostic thresholds and therefore can also capture subtle changes that happen below, or above, that concentration. We recommend that new standardised bioassays are developed that produce consistent dose-mortality estimates with a minimal number of mosquitoes required.
